# Luminal Bioavailability of Orally Administered ω-3 PUFAs in the Distal Small Intestine, and Associated Changes to the Ileal Microbiome, in Humans with a Temporary Ileostomy

**DOI:** 10.1093/jn/nxab113

**Published:** 2021-05-24

**Authors:** Gael Nana, Suparna Mitra, Henry Watson, Caroline Young, Henry M Wood, Sarah L Perry, Amanda D Race, Philip Quirke, Giles J Toogood, Paul M Loadman, Mark A Hull

**Affiliations:** Leeds Institute of Medical Research, St James's University Hospital, University of Leeds, Leeds, United Kingdom; Department of Hepatobiliary Surgery, St James's University Hospital, Leeds Teaching Hospitals NHS Trust, Leeds, United Kingdom; Leeds Institute of Medical Research, St James's University Hospital, University of Leeds, Leeds, United Kingdom; Leeds Institute of Medical Research, St James's University Hospital, University of Leeds, Leeds, United Kingdom; Department of Hepatobiliary Surgery, St James's University Hospital, Leeds Teaching Hospitals NHS Trust, Leeds, United Kingdom; Leeds Institute of Medical Research, St James's University Hospital, University of Leeds, Leeds, United Kingdom; Leeds Institute of Medical Research, St James's University Hospital, University of Leeds, Leeds, United Kingdom; Leeds Institute of Medical Research, St James's University Hospital, University of Leeds, Leeds, United Kingdom; Institute of Cancer Therapeutics, University of Bradford, Bradford, United Kingdom; Leeds Institute of Medical Research, St James's University Hospital, University of Leeds, Leeds, United Kingdom; Department of Hepatobiliary Surgery, St James's University Hospital, Leeds Teaching Hospitals NHS Trust, Leeds, United Kingdom; Institute of Cancer Therapeutics, University of Bradford, Bradford, United Kingdom; Leeds Institute of Medical Research, St James's University Hospital, University of Leeds, Leeds, United Kingdom

**Keywords:** docosahexaenoic acid, eicosapentaenoic acid, ileostomy, omega-3 polyunsaturated fatty acid, small intestine

## Abstract

**Background:**

Oral administration of purified omega-3 (ω-3) PUFAs is associated with changes to the fecal microbiome. However, it is not known whether this effect is associated with increased PUFA concentrations in the gut.

**Objectives:**

We investigated the luminal bioavailability of oral ω-3 PUFAs (daily dose 1 g EPA and 1g DHA free fatty acid equivalents as triglycerides in soft-gel capsules, twice daily) and changes to the gut microbiome, in the ileum.

**Methods:**

Ileostomy fluid (IF) and blood were obtained at baseline, after first capsule dosing (median 2 h), and at a similar time after final dosing on day 28, in 11 individuals (median age 63 y) with a temporary ileostomy. Fatty acids were measured by LC–tandem MS. The ileal microbiome was characterized by 16S rRNA PCR and Illumina sequencing.

**Results:**

There was a mean 6.0 ± 9.8-fold and 6.6 ± 9.6-fold increase in ileal EPA and DHA concentrations (primary outcome), respectively, at 28 d, which was associated with increased RBC ω-3 PUFA content (*P* ≤ 0.05). The first oral dose did not increase the ileal ω-3 PUFA concentration except in 4 individuals, who displayed high luminal EPA and DHA concentrations, which reduced to concentrations similar to the overall study population at day 28, suggesting physiological adaptation. *Bacteroides, Clostridium*, and *Streptococcus* were abundant bacterial genera in the ileum. Ileal microbiome variability over time and between individuals was large, with no consistent change associated with acute ω-3 PUFA dosing. However, high concentrations of EPA and DHA in IF on day 28 were associated with higher abundance of *Bacteroides* (*r*^2^ > 0.86, *P* < 0.05) and reduced abundance of other genera, including *Actinomyces* (*r*^2^ > 0.94, *P* < 0.05).

**Conclusions:**

Oral administration of ω-3 PUFAs leads to increased luminal ω-3 PUFA concentrations and changes to the microbiome, in the ileum of individuals with a temporary ileostomy. This study is registered on the ISRCTN registry as ISRCTN14530452.

## Introduction

Omega-3 (ω-3) PUFAs are licensed for treatment of severe hypertriglyceridemia and prevention of cardiovascular events in high-risk individuals (1). ω-3 PUFAs are also widely used as a nutritional supplement, either in complex “fish oil” mixtures or, more commonly, in concentrated “nutraceutical” forms (2).

We have reported that oral administration of 4 g of a 1:1 mix of the 2 main marine-derived ω-3 PUFAs, namely EPA (20:5ω-3) and DHA (22:6ω-3), daily for 8 wk is associated with a reversible change in the fecal microbiome in middle-aged volunteers (3). ω-3 PUFA use was associated with increased abundance of SCFA-producing bacterial taxa such as *Lactobacillus, Roseburia*, and *Sutterella* (3). Oral administration of EPA 2 g daily reduces rectal adenoma number and size in patients with familial adenomatous polyposis (4) and decreases “sporadic” conventional colorectal adenoma risk in the distal colon and rectum (5), which is consistent with the hypothesis that anti–colorectal cancer (CRC) activity of ω-3 PUFA is, at least in part, mediated by increased luminal concentrations of SCFAs, the receptors for which are expressed predominantly in the distal colon (6). Others have also now demonstrated that ω-3 PUFA use is associated with changes to the human fecal microbiome (7, 8).

It is not known whether changes to the gut microbiome, which are associated with oral intake of ω-3 PUFAs, are mediated by increased exposure of the gut microbiota to intestinal luminal ω-3 PUFAs. Moreover, it is not known whether ω-3 PUFAs detected in the distal digestive tract lumen derive directly from oral dosing or indirectly via the intestinal mucosal surface following absorption in the proximal small bowel and widespread tissue incorporation throughout the body. One study has measured ω-3 PUFA recovery (as the percentage of the oral dose) in ileostomy effluent after dosing with a single 1000-mg fish oil capsule (containing 48 mg EPA and 218 mg DHA) taken with water (9). This study reported that <1% of the total ω-3 PUFAs administered were recovered in the ileostomy effluent between 2 and 16 h after single dosing (9). This is consistent with the current dogma that efficient PUFA absorption occurs in the proximal small bowel, regardless of the chemical form of the PUFAs (as free fatty acids, ethyl ester conjugates, or triglycerides), particularly if ω-3 PUFA dosing occurs with food (10). However, the dose of EPA and DHA used in the Australian study (9) was far lower than those associated with effects on the gut microbiota and colorectal neoplasia in prior clinical studies (which have used 2–4 g daily) (3–5, 11). Moreover, the authors did not report PUFA concentrations in ileal effluent, which are needed to investigate properly the interactions between PUFAs and gut bacteria in ex vivo intestinal models.

Therefore, we studied the intestinal luminal and systemic bioavailability of EPA and DHA in individuals with a temporary ileostomy undergoing oral dosing with 4 g daily of a 1:1 mix of EPA and DHA for 28 d. We compared PUFA concentrations obtained in the distal ileum [also acting as a proxy for proximal colonic concentrations (12)] acutely (<24 h after the first dose) and after longer-term oral dosing (at 28 d). We also investigated the relation between intestinal ω-3 PUFA concentrations and changes to the luminal microbiome in the ileum.

## Methods

This study was conducted according to the Declaration of Helsinki, and all procedures involving human subjects/patients were approved by the Yorkshire & Humber-Leeds West Research Ethics Committee (15/YH/0547). Written informed consent was obtained from all subjects. The study was registered in a publicly available trials registry (ISRCTN14530452). The intervention was not classified as Investigational Medicinal Product by the UK Medicines and Healthcare Products Regulatory Agency.

### Human subjects

We recruited individuals, aged ≥18 y, of either sex, who had a temporary loop ileostomy, which had been fashioned during anterior resection for CRC ≥2 mo prior to enrolment, for whom eventual ileostomy reversal was planned ≥2 mo later. The original eligible age threshold was 50 y (stipulated so that the age range of participants mirrored a population at risk of colorectal neoplasia). However, due to slow recruitment, we amended that eligibility criterion (with Research Ethics Committee substantial amendment) so that the lower age limit was 18 y. We excluded individuals who: *1*) were using (at any time within 4 wk of recruitment) an ω-3 PUFA–containing supplement (including cod liver oil); *2*) had received any form of systemic chemotherapy or radiotherapy in the past 4 wk; *3*) had known metastatic CRC; *4*) had small bowel disease such as Crohn disease or celiac disease; *5*) had previously undergone any small bowel resection >10 cm; or *6*) were allergic to any type of seafood.

Suitable patients were screened from colorectal surgery department records at St James's University Hospital, Leeds, United Kingdom, and sent a participant information leaflet. Interested individuals attended the hospital for a detailed eligibility check and then provided written informed consent, if appropriate.

**FIGURE 1:**
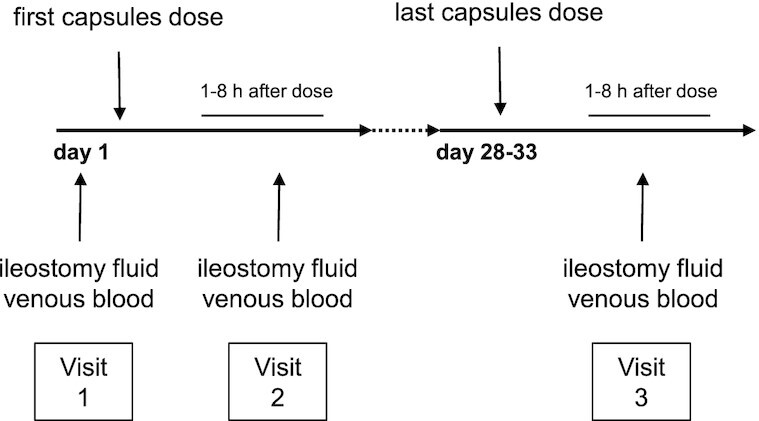
Schedule of ω-3 PUFA capsules dosing and biosample collection from patients with a temporary ileostomy. Ileostomy fluid and blood were obtained before the first capsules were taken (visit 1) and 1–8 h after the first capsules dose (visit 2), as well as 1–8 h after the last capsules dose (visit 3), which was after 28 d of regular ω-3 PUFA capsule dosing. ω-3 PUFA capsules were always taken with meals.

Participants undertook 3 further study visits over a 28-d period (**Figure 1**), with visit 1 coinciding with a hospital visit for routine care, when at all possible. Open-label ω-3 PUFA capsules were issued to patients after written informed consent was obtained.

### The ω-3 PUFA intervention

Participants were instructed to take 2 soft-gel capsules (EuroCaps Ltd), each containing 481 mg EPA-triglyceride and 481 mg DHA-triglyceride (500 mg/g) concentrates (Incromega; Croda Ltd), twice daily with meals for 28 d, thereby providing a daily dose of 1 g EPA free fatty acid equivalent and 1 g DHA free fatty acid equivalent.

### Study visit schedule

At visit 1, participants provided a pretreatment (baseline) ileostomy fluid (IF) sample, in addition to a venous blood sample, prior to taking the first dose of capsules (Figure 1). A second IF sample and blood sample were obtained at visit 2, which occurred at a time and place (the hospital or at home) directed by the participant, ≥1 h and ≤8 h after taking the first ω-3 PUFA capsules dose with a meal. After 4 wk of twice-daily dosing with oral ω-3 PUFA capsules, participants were required to provide a final IF and blood sample at visit 3 at an identical time (±1 h) after the last ω-3 PUFA capsules dose as the duration between first capsule dosing and sampling at visit 2. A 24-h ileostomy output collection was deemed to be unacceptable for participants, after taking advice from a patient representative. Therefore, we collected data on the frequency of stoma bag emptying over the preceding 24 h as a proxy for the ileostomy output volume. At each visit, participants were asked about the presence of gastrointestinal symptoms, and any other adverse events that had occurred during the intervention period were recorded at visit 3. The number of capsules returned at visit 3 was used as an indicator of compliance.

### Sample collection and analysis

Venous blood was collected into 2 EDTA-containing tubes, which were centrifuged at 800 × *g* for 5 min at 4°C within 30 min of collection. Plasma and RBCs were separated, aliquoted, and stored at −80°C until analysis.

IF samples were aliquoted into 2 sterile 20-mL plastic containers, held on ice, and stored at −80°C before analysis.

### LC–tandem MS measurement of fatty acids

Ileostomy content (150 mg) was added to 3 mL distilled water and homogenized by vortex mixing for 60 s. Lipids were extracted from 100 μL of the ileostomy homogenate as described (13). RBCs (50 μL) were diluted 1:1 with 50 μL distilled H_2_O before lipid extraction. In every sample, 4 μL of internal standard (50 μg/mL α-linolenic acid-d_14_) was added (13).

Fatty acids were measured by LC–tandem MS (13). RBC ω-3 PUFA concentrations are presented as the relative amount (percentage total fatty acids). ω-3 PUFA concentrations in plasma and IF are reported as the absolute concentration in micrograms per milliliter.

### Measurement of IF osmolality

IF was centrifuged at 3000 × *g* for 10 min at room temperature to produce a clear supernatant for measurement of osmolality by freezing point depression osmometry using an Advanced 2020 Multi-Sample Osmometer (Advanced Instruments).

### Ileal microbiome analysis

DNA was extracted from ∼200 mg frozen IF using a modified version of the QIAamp DNA Stool Mini Kit protocol (QIAGEN) with bead-beating. DNA was quantified by NanoDrop spectrophotometry. DNA underwent PCR amplification of the V4 region of the 16S rRNA gene and library preparation according to the Earth Microbiome Project 16S Illumina Amplicon methodology (http://www.earthmicrobiome.org) with single PCR reactions per sample [515F (Parada) 5´-GTGYCAGCMGCCGCGGTAA-3´ and 806R (Apprill) 5´-GGACTACNVGGGTWTCTAAT-3´]. Sequencing was performed on an Illumina HiSeq 3000 2 × 150-bp paired end lane. Reads were stripped of adapter sequences using Cutadapt (v1.18) (14). Initial analysis was performed in the QIIME2 environment (v2019.10) (15). Reads were shortened to a maximum of 145 bp, merged, denoised, and representative sequences picked using DADA2 (16). Representative reads were aligned to the SILVA database (release 132) using the BLAST+ algorithm (17, 18). All taxonomy assignments were exported to MEGAN6 for further analysis (19), which was focused on level 6 (genus) assignments.

### Statistical analysis

There are no data on absolute ileal or fecal EPA and DHA concentrations generated during oral ω-3 PUFA supplement use. Therefore, we were unable to perform a formal sample size calculation based on an a priori coprimary end point of the study (ileal ω-3 EPA and DHA concentrations at the end of the treatment period). Previous studies of ω-3 PUFA RBC membrane incorporation associated with supplementation have reported large intraindividual (4%) and interindividual (≤12%) CVs (9, 20). Based on a mean molar percentage plasma EPA concentration of 0.4 reflecting ileal EPA content at baseline and the above interindividual variance (21), we calculated that 10 participants would detect a minimum 30% increase in ileal EPA concentration with 80% power at a 5% significance level, the recruitment of which would be feasible in a 1-y study period with a maximum expected recruitment rate of 10% for an experimental intervention study in patients undergoing 2-stage cancer surgery (102 patients underwent temporary ileostomy formation at St James's University Hospital during June 2014 to May 2015). The predicted sample size was larger than the only previous clinical study of ileal ω-3 PUFA content (9).

Given the small size of this human intervention study, all other analyses (including relative ω-3 PUFA concentrations in RBC membranes, other ileal fluid measurements, and ileal microbiome analysis) were classified as exploratory with hypothesis testing using α = 0.05, acknowledging the increased risk of type I statistical error.

Statistical analysis and graphical representation of the PUFA data was performed using GraphPad Prism version 8 (GraphPad Software, Inc) and SPSS version 26 (IBM, Inc). Longitudinal intraindividual data were compared using a 2-sided paired Student *t* test. Student unpaired *t* test was used to compare values between subgroups of participants, including sensitivity analysis of individuals with and without an early peak in ileal EPA and DHA concentration. Nonparametric tests (Mann–Whitney *U* test and Wilcoxon signed-rank test) were also used for unpaired and paired group analyses, for data with a nonnormal distribution (Kolmogorov–Smirnov normality test; *P* < 0.05). The Spearman rank correlation test was used to test the relation between ω-3 PUFA concentrations and timing of sampling. In all cases, a *P* value <0.05 was deemed statistically significant.

Statistical analysis of the ileal microbiome data was performed using the R package (22). β Diversity between visit samples and across participants was tested using the Adonis permutational multivariate ANOVA (PERMANOVA) test. Ileal microbiome (Pearson) correlation analyses were all corrected for multiple comparisons using the Benjamini–Hochberg false discovery rate procedure.

## Results

**FIGURE 2:**
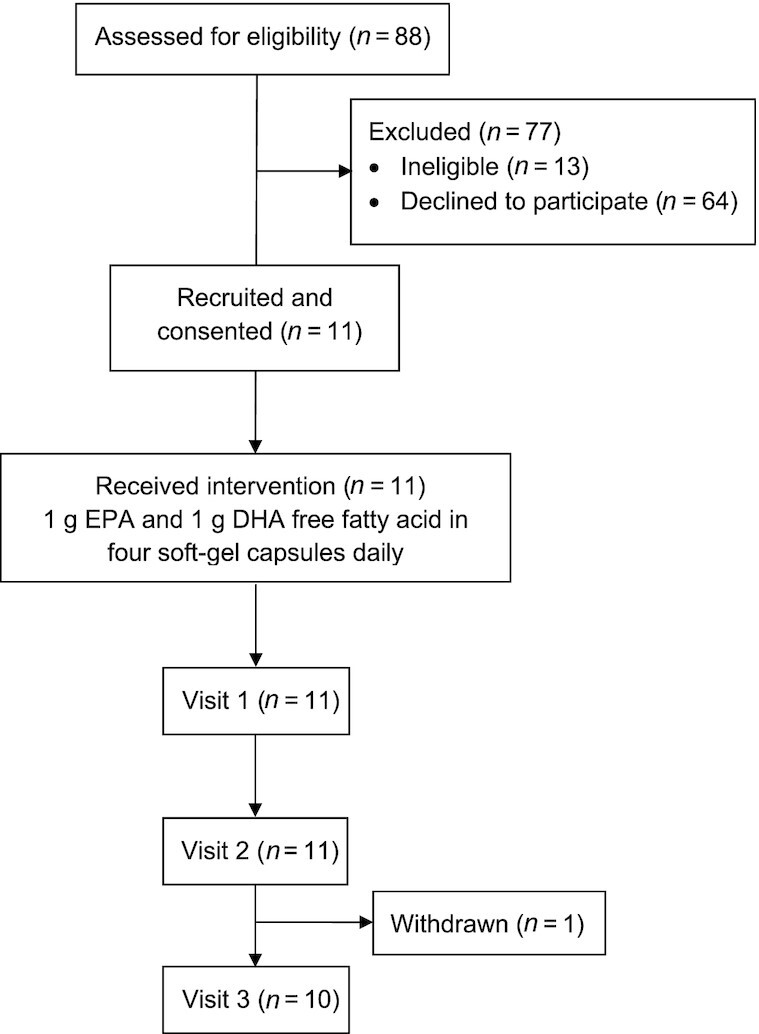
Participant flow diagram. Eight individuals completed the study per protocol.

Eleven individuals with a temporary loop ileostomy were recruited to the study between April 2016 and December 2018 after screening 88 patients (**Figure 2**). Demographic and clinical details are presented in **Table 1**. Eight individuals completed the study per protocol, thus providing complete sample sets during ω-3 PUFA capsules dosing. One participant was withdrawn from the study after visit 2 (Figure 2) when it was realized that the individual was ineligible due to age (42 y when the eligibility criterion was >50 y). That individual had provided consent for the data collected to date to be used after withdrawal. One participant stopped taking capsules on day 24 after reporting several gastrointestinal adverse events (eructation, nausea and abdominal discomfort), which did not improve after dose reduction. One blood sample was not obtained at visit 2 due to failed venipuncture. Compliance with capsule dosing was excellent as assessed by capsule counting (Table 1). There were no other adverse events reported during ω-3 PUFA capsules dosing for 4 wk during the study.

**TABLE 1:** Participant characteristics^1^

Age, y	63 (42–81)
Sex (male:female)	8:3
Duration of temporary ileostomy use before the study, mo	9 (2–18)
Frequency of ileostomy bag emptying in the last 24 h	
Visit 1	3 (1–7)
Visit 3	4 (1–7)
Time between last oral dose and sample collection, h	
Visit 2	2 (1–4)
Visit 3	2 (1–5)
Duration of ω-3 PUFA capsule intervention, d	28 (1–33)
Capsule compliance,^2^ %	100 (50–107)

1 Data are the median value and range of values, unless stated otherwise.

2 Capsule compliance calculated as: observed number of capsules taken [168 (number of capsules given to each participant) − number of capsules returned] ÷ expected number of capsules taken (4 × number of days taking capsules) × 100.

**FIGURE 3:**
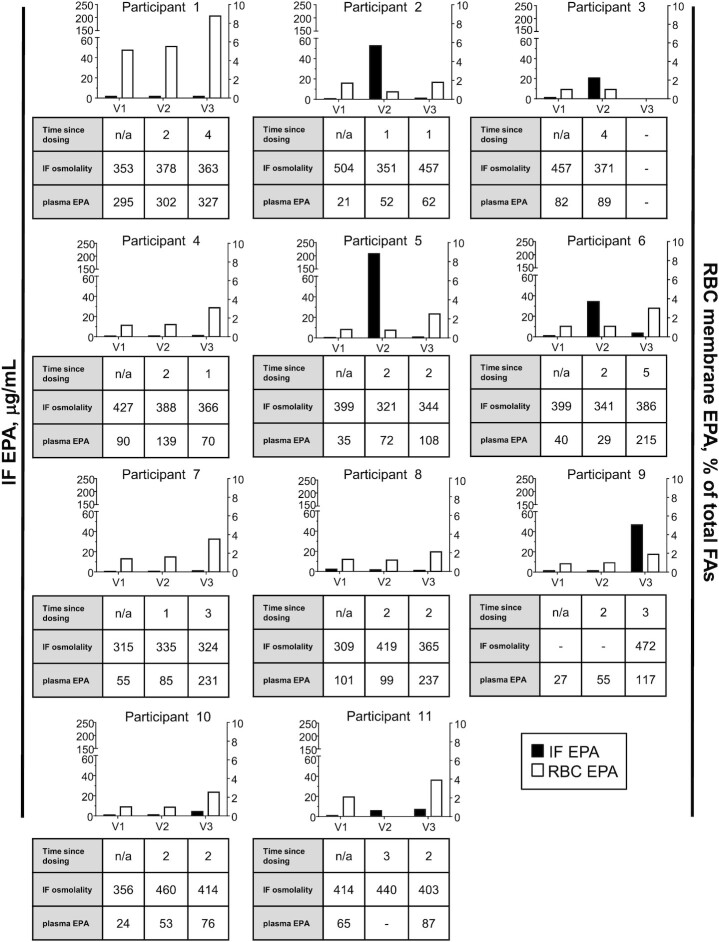
Ileostomy fluid and RBC membrane EPA concentrations in individual participants with a temporary ileostomy before and after ω-3 PUFA capsule dosing. The concentration of EPA in ileostomy fluid (IF) and the EPA content of RBC membranes at each time point for every participant is shown. Corresponding data about the timing of sample collection for V2 and V3 samples in relation to ω-3 PUFA capsules dosing (in hours), osmolality of IF (in milliosmoles per kilogram), and the plasma EPA concentration (micrograms per milliliter) for all visit samples are presented below each figure panel. FA, fatty acid; IF, ileostomy fluid; V, visit.

**FIGURE 4:**
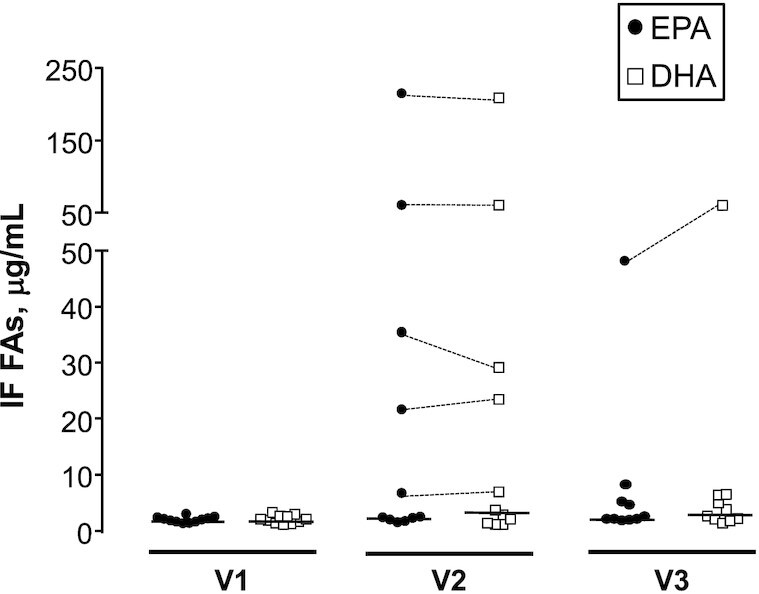
The distribution of ileostomy fluid EPA and DHA values in individual participants with a temporary ileostomy. Individual data points are presented for both EPA and DHA. The bar denotes the median value for each group. Dotted lines join EPA and DHA values from those individuals with higher ω-3 PUFA concentrations at V2 and V3. In each case, there was a consistently high concentration of both EPA and DHA in keeping with the 1:1 ratio of EPA and DHA in the oral ω-3 PUFA capsule intervention. FA, fatty acid; IF, ileostomy fluid; V, visit.

Individual participant IF, RBC, and plasma EPA profiles during the study are presented in **Figure 3**. Equivalent data for DHA are available as **Supplemental Figure 1**. Comparative ω-3 PUFA data are presented in **Figure 4**. In all cases, there was <5 μg/mL EPA and DHA present in IF at baseline prior to oral ω-3 PUFA dosing. Following the first dose of EPA/DHA with a meal (median 2 h later), 4 participants exhibited an increase in EPA and DHA content of ileal content (participants #2, #3, #5, and #6), whereas the majority of participants (participants #1, #4, and #7–11) displayed no clear increase in ileal ω-3 PUFA concentrations (Figure 3, Supplemental Figure 1). In every case, a large rise in the concentration of EPA after initial ω-3 PUFA capsules dosing (at visit 2) was accompanied by a similar rise in DHA concentration (Figure 4). The acute rise in EPA and DHA in a subgroup of 4 individuals was not explained by a difference between the 2 groups of individuals regarding the duration between dosing and IF sample collection [mean = 2 h (range = 1–4 h) compared with 2 h (range = 1–3 h); *P* = 1.0, Mann–Whitney *U* test]. Overall, there was no relation between the ileal EPA or DHA concentration and duration between oral dosing and sample collection at visit 2 (ρ = 0.20, *P* = 0.57 and ρ = −0.17, *P* = 0.61, respectively; Spearman rank correlation). The increase in EPA and DHA content of IF in the subgroup of 4 participants was also not due to more concentrated ileostomy output based on the osmolality of the IF, which remained similar across the study visits and was actually lower in the visit 2 samples that contained higher concentrations of EPA and DHA (mean = 346 ± 21 compared with 403 ± 45 mOsm/kg, *P* = 0.05; Student unpaired *t* test; Supplemental Figure 1, Figure 3). We hypothesized that the appearance of EPA and DHA in the ileum in 4 participants was due to lower absorption in the proximal small intestine, which might be associated with an absence of a peak in plasma ω-3 PUFA concentrations after oral dosing. However, there was no difference in peak plasma EPA (mean = 61 ± 26 compared with 122 ± 97 μg/mL; *P* = 0.24; Student unpaired *t* test) or peak plasma DHA (mean = 74 ± 35 compared with 90 ± 45 μg/mL; *P* = 0.55) concentrations at visit 2, or the absolute difference in concentration at visit 2 of EPA (16 ± 22 compared with 24 ± 18 μg/mL; *P* = 0.57) or DHA (−15 ± 84 compared with 29 ± 31 μg/mL; *P* = 0.27) compared with baseline values, between those individuals with an increase in ileal EPA/DHA (participants #2, #3, #5, and #6) and those with stable low concentrations of ω-3 PUFAs (participants #1, #4, and #7–11) after first oral supplementation (Supplemental Figure 1, Figure 3). However, the lack of an acute peak plasma ω-3 PUFA response to oral dosing is likely due to insufficient time between dosing and plasma collection in this study (23).

In contrast to the rapid changes in EPA and DHA content in IF after the first dose of ω-3 PUFA capsules in 4 participants, there was no consistent change in concentration of the other fatty acids that were measured at this time point (**Supplemental Table 1**). Notably, there was no consistent increase in IF docosapentaenoic acid (DPA) concentration after first dosing (*P* = 0.21 for the difference between visit 1 and visit 2 concentrations; paired *t* test), even in those participants with an acute rise in EPA and DHA concentrations at visit 2 (0.04 ± 0.03 μg/mL for participants #1, #4, and #7–11 compared with 0.29 ± 0.34 μg/mL for participants #2, #3, #5, and #6; *P* = 0.08; Student unpaired *t* test), in keeping with direct transport of orally administered EPA and DHA via the intestinal lumen (Supplemental Table 1).

As expected, ∼4 wk of oral dosing with 4 g ω-3 PUFA triglycerides led to a consistent increase in EPA and DHA content in RBC membranes, with a mean EPA and DHA content of RBCs at 4 wk of 3.3 ± 2.0% (baseline 1.6 ± 1.2%; *P* < 0.01; paired *t* test) and 7.2 ± 2.7% (baseline 5.5 ± 3.2%; *P* = 0.05; paired *t* test), respectively (Supplemental Figure 1, Figure 3), which is consistent with previous data on RBC incorporation of ω-3 PUFAs in different human populations (24, 25). The RBC data were mirrored by a statistically significant increase in plasma EPA and DHA concentrations in visit 3 samples compared with baseline (visit 1) values (*P* = 0.01 and *P* < 0.01, respectively; paired *t* test; Supplemental Figure 1, Figure 3). This supports the notion that there was excellent capsule compliance and that the physiology of ω-3 PUFA intestinal absorption in participants with a temporary loop ileostomy is relevant to individuals with an intact gastrointestinal tract.

Of note, in the 3 individuals for which there was a visit 3 IF sample (participants #2, #5, and #6), the increased concentration of EPA and DHA in ileal content at visit 2 had returned to concentrations (mean = 1.8 ± SD 1.5 and 2.3 ± 1.8 μg/mL, respectively) consistent with the other participants, who did not have a visit 2 “peak” (9.0 ± 16.9 and 9.5 ± 18.7 , respectively; both *P* = 0.5, Student unpaired *t* test), despite the fact that the time between oral dosing and sample collection was similar between visit 2 and visit 3. This is consistent with a possible adaptive response to oral ω-3 PUFA dosing in individuals who initially exhibited increased distal intestinal EPA and DHA bioavailability after first oral ω-3 PUFA dosing.

Overall, baseline (visit 1) IF EPA and DHA concentrations increased from a mean of 1.00 ± 0.55 μg/mL (*n* = 10) and 0.90 ± 0.68 μg/mL, respectively, to 6.88 ± 14.28 μg/mL (*P* = 0.025, Wilcoxon signed-rank test) and 7.34 ± 15.69 μg/mL (*P* = 0.008, Wilcoxon signed-rank test) after 28 d of oral dosing (visit 3), which represents a mean fold increase of 6.0 ± 9.8 for EPA and 6.6 ± 9.6 for DHA in IF samples after oral supplementation with ω-3 PUFAs. Excluding those individuals who had a reversible peak in EPA and DHA concentrations at visit 2 (participants #2, #3, #5, and #6), there was an increase in IF EPA and DHA concentration at visit 3 compared with the corresponding visit 2 value, but, in both cases, the difference failed to reach statistical significance (*P* = 0.063 and 0.11 for EPA and DHA, respectively; Wilcoxon signed-rank test). Consistent with the visit 2 data, there was also no relation between the ileal EPA or DHA concentration and duration between oral dosing and sample collection at visit 3 (ρ = 0.36, *P* = 0.30 and ρ = −0.26, *P* = 0.46, respectively; Spearman rank correlation).

The human small intestinal microbiota has been investigated inadequately in comparison with the colonic (fecal) microbiota, primarily related to the relative difficulty with which human small intestinal luminal samples can be obtained (26). Therefore, we took the opportunity to investigate the effects of oral ω-3 PUFA supplementation on the small intestinal microbiome in individuals with a temporary ileostomy.

**FIGURE 5:**
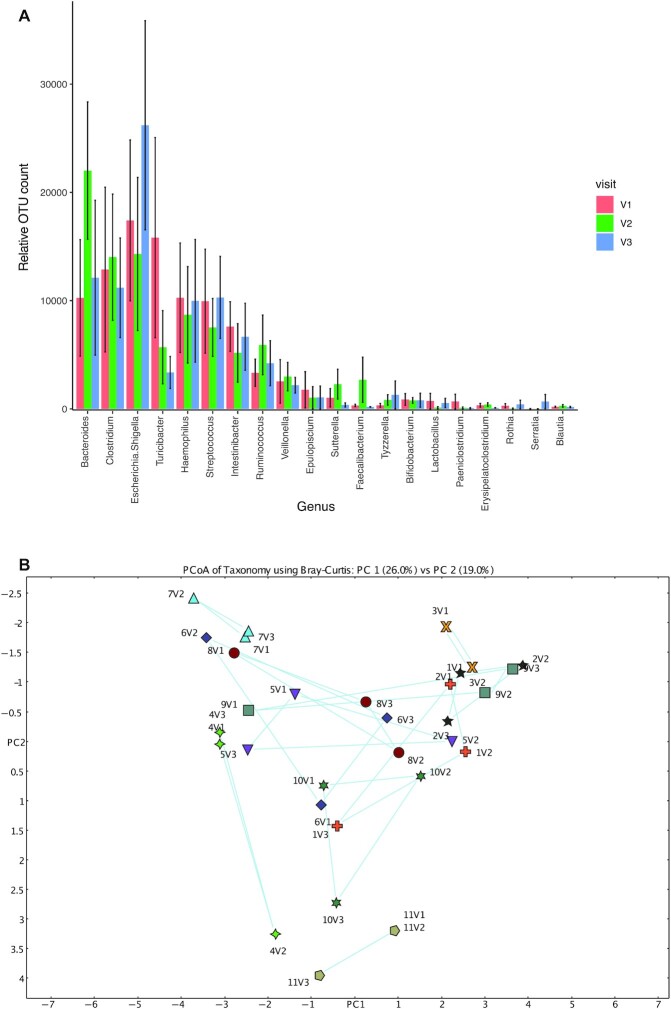
Analysis of the ileal microbiome before, during, and at the end of oral ω-3 PUFA supplementation in patients with a temporary ileostomy. (A) The 20 most abundant bacterial genera in ileostomy fluid at baseline, as well as during and after oral dosing with ω-3 PUFAs for 28 d. Columns represent the mean relative OTU counts for all participants who provided the relevant sample at baseline (V1; red), after the first dose of ω-3 PUFA capsules (V2; green) and after 28 d of oral ω-3 PUFA dosing (V3; blue). Bars represent the SEs of the mean. (B) Bray–Curtis PCoA of the ileal microbiome before and after oral dosing with ω-3 PUFAs. Each participant is represented by a symbol of different color and shape, as well as a participant number label. Blue lines join data points at baseline (V1), after the first dose of ω-3 PUFA capsules (V2), and after the last dose of ω-3 PUFA capsules (V3) after 28 d of dosing with oral ω-3 PUFA capsules. OTU, operational taxonomic unit; PC, principal component; PCoA, principal component analysis; V, visit.

Consistent with previous reports describing the bacterial structure of ileal fluid, we found that the dominant genera in ileal content prior to ω-3 PUFA supplementation consisted of *Bacteroides, Clostridium, Escherichia/Shigella, Turcibacter, Haemophilus*, and *Streptococcus* (**Supplemental Figure 2**, **Figure 5**A) (25). QIIME2 combined the 2 *Enterobacteriaceae* genera *Escherichia* and *Shigella* as a single genus category, which was then used in MEGAN for further analysis. The top 10 genera made up >90% of the total operational taxonomic units (OTUs) measured (Figure 5A). As expected, there was wide variation in the ileal microbiome between individuals at baseline (visit 1) demonstrated by Bray–Curtis principal component analysis (PCoA; SupplementalFigure 2, Figure 5B). Consistent with the dynamic nature of small intestinal content and its microbiota, there were large intraparticipant differences between the baseline IF sample (visit 1) and the corresponding sample taken a few hours after first oral dosing with ω-3 PUFA capsules (visit 2; Figure 5B). There was no significant difference across the visit profiles (PERMANOVA, *P* = 0.93) with variability between the participants being the only significant variable (PERMANOVA, *P* = 0.001). Importantly, there was no discernible difference in the relation between visit 1 and visit 2 profiles between individuals who displayed an EPA and DHA peak after first oral dosing (participants #2, #3, #5, and #6) and those that did not (participants #1, #4, and #7–11). This suggests that an acute increase in luminal EPA and DHA concentration does not acutely change the ileal microbiome in a consistent manner, over and above natural, dynamic variability in ileal content over short periods of time (Figure 5B). Consistent with this notion, the ileal microbiome in the participants who displayed a visit 2 EPA and DHA peak did not return consistently to a microbiome profile at visit 3 more closely resembling visit 1, even though IF EPA and DHA concentrations consistently reduced to concentrations of the same order of magnitude as visit 1 values in all participants (Figure 5B).

In keeping with the large intra- and interindividual variability in the ileal microbiome demonstrated by PCoA, there were major shifts in abundance of the top 10 genera between the 3 samples, before (visit 1), during (visit 2), and immediately after (visit 3) ω-3 PUFA dosing (Figure 5A). Changes in the top 15 most abundant genera after the first dose of ω-3 PUFA capsules (difference between visit 2 and visit 1) and after oral ω-3 PUFA supplementation therapy for 28 d (difference between visit 3 and visit 1) in each of the participants are represented in Supplemental Figure 2. There was no consistent relation observed between the difference in genus abundance associated with first dosing with ω-3 PUFA capsules and that associated with dosing for 28 d in any of the participants (Supplemental Figure 2). There was also no evidence that changes in taxa abundance at each of the 2 time points compared with baseline were common across all the participants, although *Bacteroides* and *Veillonella* abundance was static or increased in 8 of the 11 participants across both time points (Supplemental Figure 2).

**FIGURE 6:**
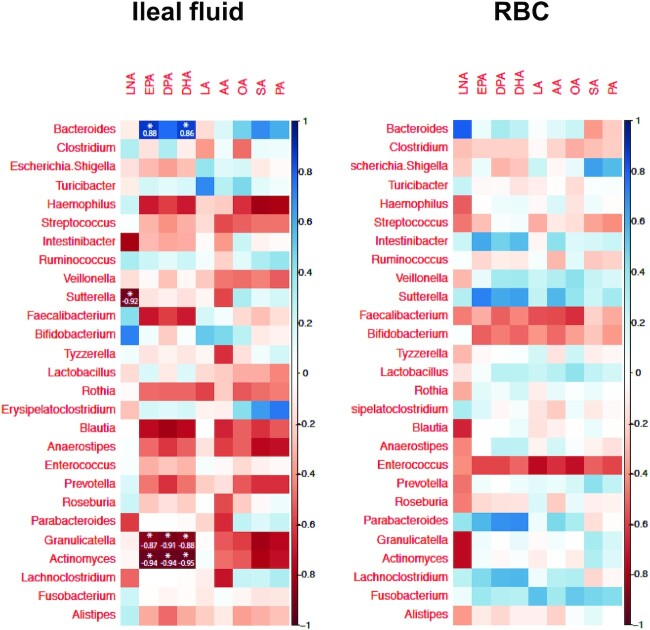
Correlation matrices for the difference in bacterial genus abundance and the difference in absolute ileal fluid fatty acid concentrations (and RBC percentage content) between baseline (V1) and after 28 d of treatment with ω-3 PUFA capsules (V3) in patients with a temporary ileostomy. Blue denotes a positive correlation. Red denotes a negative correlation. The strength of the Pearson correlation is denoted by the colour intensity (side-bar scale). Statistically significant (*P* < 0.05) relations are signified by an asterisk with the actual *r*^2^ value. AA, arachidonic acid; DPA, ω-3 docosapentaenoic acid; LA, linoleic acid; LNA, α-linolenic acid; OA, oleic acid; PA, palmitic acid; SA, stearic acid, V, visit.

Next, we explored the relation between genus abundance in ileal content and ileal fluid fatty acid concentrations in each participant. Eight of the top 35 genera had zero OTU abundance in >5 samples. Therefore, we decided to exclude those taxa from correlation analysis. There was a strong correlation between the increase in EPA and DHA (and the direct EPA elongation metabolite DPA) concentration in ileal fluid obtained after 28 d dosing with ω-3 PUFAs (visit 3) compared with baseline (visit 1) and the change in abundance of several genera between the same time points (**Figure 6**). Higher concentrations of the supplemented ω-3 PUFAs in ileal fluid were associated with an increased abundance of *Bacteroides*, which was not observed for the other fatty acids including the other ω-3 PUFA, 18:3ω-3 α-linolenic acid (LNA; Figure 6). Conversely, increased concentrations of EPA and DHA in ileal fluid were associated with reduced abundance of 2 genera (*Granulicatella* and *Actinomyces*), which was not shared by the other fatty acids that were measured (Figure 6). In contrast, changes in luminal concentrations of the shorter-chain-length ω-3 PUFA that was not supplemented (LNA) correlated with different genera in ileal fluid including *Sutterella* (Figure 6). Importantly, no significant correlations were observed between the change in bacterial abundance between baseline to visit 3 and fatty acid concentrations in RBC membranes (Figure 6), suggesting that the relations between altered abundance of specific genera and concentrations of supplemented ω-3 PUFAs are specific to the local ileal environment, not an increase in systemic concentrations of EPA and/or DHA. A similar analysis was also performed to explore the relation between genus abundance in ileal content and ileal fluid fatty acid concentrations after acute dosing at visit 2 (after the first capsules dose). In contrast to the relations observed between the change in EPA and DHA concentrations in the ileum and bacterial abundance after oral dosing for 28 d (Figure 6), there was no evidence of an association between the acute change in either EPA or DHA concentrations and bacterial abundance in ileal fluid (**Supplemental Figure 3**). This confirms that acute changes in ileal ω-3 PUFA concentrations do not alter the ileal microbiome, unlike oral supplementation with EPA and DHA for several weeks. There were 4 isolated statistically significant negative correlations between the difference in RBC content of fatty acids, including oleic acid, and 3 bacterial genera after first dosing, which lack biological plausibility given the kinetics of fatty acid incorporation into RBC membranes and could have resulted from multiple testing (Supplemental Figure 3).

## Discussion

We report that twice daily oral supplementation with EPA and DHA for 28 d (providing the equivalent of 1 g EPA free fatty acid and 1 g DHA free fatty acid per day) leads to an increase in ileal fluid EPA and DHA content, which is associated with an increased abundance of *Bacteroides* and a decrease in other bacterial genera that is observed despite high variability in the distal small bowel microbiota between individuals and over time.

Assuming a 24-h ileostomy output of 1000 mL, one can estimate that an approximate EPA and DHA concentration of 7 μg/mL in ileal fluid after 28 d dosing could equate to as little as 1% of the orally administered dose appearing in the ileum (and hence the proximal colon) assuming that all luminal ω-3 PUFAs derive directly from oral administration, rather than indirectly from small intestinal mucosa. This is a similar magnitude to the proportion of ω-3 PUFAs recovered in ileal fluid after oral administration of a smaller dose (<300 mg) of ω-3 PUFAs (<1%) in a previous study (9). Consistent with equal dosing with EPA and DHA in this study, similar changes in luminal concentrations of EPA and DHA were measured in each participant. As expected, there were no other significant changes in the fatty acid profile of ileal fluid after oral administration of pure EPA and DHA. Knowledge that microgram per milliliter concentrations of EPA and DHA are generated in the distal small intestine after oral dosing will inform ex vivo experiments investigating EPA- and DHA-derived bioactive lipid mediator production and activity on the gut microbiota, including proresolving oxylipins such as E- and D-type resolvins (27).

We designed our study so that we compared ileal fluid samples after the first dose of ω-3 PUFA capsules and after the last dose of capsules (after supplementation for 28 d) at an equivalent time after ingestion. This allowed us to estimate the contribution of direct oral delivery of ω-3 PUFAs to ileal ω-3 PUFA bioavailability (visit 2 concentrations in previously ω-3 PUFA–“naïve” ileal fluid) compared with delivery of ω-3 PUFAs to the ileal lumen via the ileal mucosa—hypothesized to occur only after medium-term (28-d) dosing (visit 3) that produces increased tissue (mucosal) ω-3 PUFA concentrations. The acute rise in EPA and DHA concentrations in ileal fluid, which was demonstrated in 4 individuals, confounds the comparison of visit 2 and visit 3 ω-3 PUFA concentrations in all participants. However, exclusion of this subgroup of individuals from this analysis confirmed that, although ileal fluid EPA and DHA concentrations were higher after medium-term oral dosing than before supplementation, there was no consistent or statistically significant increase in ileal ω-3 PUFA bioavailability after oral supplementation for 28 d compared with that after the first oral dose. This implies that ω-3 PUFAs originating from the ileal mucosa (perhaps through shed enterocytes) are likely to make only a small contribution to ileal (and hence proximal colonic) ω-3 PUFA bioavailability during prolonged dosing. We accept that other factors could explain differences between ileal fluid EPA and DHA concentrations after short- and longer-term oral supplementation, including changes to proximal small intestinal digestion and absorption of ω-3 PUFAs (as triglycerides) during consistent ω-3 PUFA use. The only experimental protocol that could directly address the precise contribution of luminal delivery compared with release from the mucosal surface to intestinal bioavailability in humans would be measurement of EPA and DHA concentrations in the intestinal lumen after parenteral administration of labeled ω-3 PUFAs.

An unexpected finding was the relatively high concentrations of EPA and DHA found in ileal fluid only a short time after first dosing with oral ω-3 PUFA capsules in 4 of the 11 study participants. We hypothesize that these individuals did not absorb orally administered ω-3 PUFAs to the same degree as the other participants. The reduction in EPA and DHA concentrations in ileal fluid at a similar time after dosing with capsules 28 d later suggests that physiological adaptation could then have occurred in these individuals. However, we cannot exclude that variability in the size or composition of the meal taken with the first dose of capsules explains the acute rise in luminal EPA and DHA concentrations in this subgroup. A larger study is required to quantify with greater precision the proportion of individuals that display an acute rise in intestinal ω-3 PUFA concentrations at the start of oral supplementation.

The ileal microbiome data represent a significant addition to the limited literature on the small intestinal microbiota. Historical studies using a variety of techniques have previously reported that *Bacteroides* and *Clostridium* genera are prevalent in the small intestine, which is consistent with our findings (28). Wide variability in the ileal microbiome between individuals and over time is consistent with current understanding of the dynamic nature of the small intestinal microbiota that appears reactive to nutrient flow (29). Importantly, we saw no consistent acute changes in the ileal microbiome during acute dosing, particularly in those individuals who generated relatively high EPA and DHA concentrations after first dosing. There was, however, a consistent relation between the increase in EPA and DHA content of ileal fluid and abundance of certain bacterial genera after 28 d of dosing. Of note, there was a strong relation between the increase in EPA, DPA, and DHA (but not LNA) concentrations and increased abundance of *Bacteroides* in ileal content. The *Bacteroides* genus contains many SCFA-producing species (30), which is compatible with the effect of the same daily dose of ω-3 PUFAs on SCFA-producing bacterial taxa in fecal samples from a healthy volunteer study (3) and more recent findings from a study of low-dose EPA (165 mg daily) and DHA (110 mg daily) administration on fecal *Bacteroides* abundance in healthy volunteers (8). The latter study also reported that ω-3 PUFA supplementation was associated with increased plasma concentrations of SCFAs (8). The relevance of the negative correlation between an increase in EPA and DHA concentrations and abundance of other less prominent genera such as *Actinomyces* remains unclear.

Although our data demonstrate an association between intestinal luminal concentrations of ω-3 PUFAs and abundance of some bacterial genera, we cannot infer a causal relation between ω-3 PUFA exposure and an altered ileal microbiome. Data from transgenic *Fat-1* and *Fat-2* mouse models suggest that alterations in the intestinal tissue content of ω-3 and ω-6 PUFAs modulate the fecal microbiome independently of changes in dietary PUFA intake, perhaps via a mechanism involving epithelial intestinal alkaline phosphatase expression (31, 32).

Strengths of this human intervention study include use of an ω-3 PUFA capsule formulation of pure EPA and DHA mimicking clinical trial dosing, rather than a complex PUFA mixture, thus allowing interpretation of the effects of oral supplementation on small intestinal bioavailability of the 2 main marine-derived ω-3 PUFAs without confounding by other fatty acids. Completion of a protocol whereby ileal fluid and blood sampling occurred at a similar time after dosing at the beginning and end of the intervention period was challenging, but has provided significant insight into changes in intestinal physiology and ω-3 PUFA bioavailability over a 28-d period that might have been masked if the relation with oral dosing had been less precise.

Several limitations in study design deserve attention. The size of the study was restricted by the acceptability of the experimental protocol that offered no personal benefit and mandated timed biological sampling. We accept that the presence of a temporary loop ileostomy alters small intestinal physiology, particularly increased small intestinal transit, which might explain the rapid (few hours) appearance of orally delivered EPA and DHA in ileostomy output in some participants. We deliberately stipulated a minimum period (2 mo) for any participant to have had a temporary ileostomy (in the majority, it was substantially longer) so that changes in small intestinal function during the 4-wk duration of the study were unlikely and would not be likely to explain differences in ω-3 PUFA bioavailability between acute (first dose) and chronic (after supplementation for 28 d) oral dosing. It should also be emphasized that IF can only be considered a proxy for luminal fluid in an intact terminal ileum. This is particularly relevant to the microbiome analysis, which might be confounded by contamination from the local stoma environment (26). The similarity in microbiome profile between our study of IF and other studies of ileal fluid and ileal mucosa sampled at ileocolonoscopy suggests that IF is a reasonable compromise to study the ileal microbiome without exposing study subjects to an invasive and unpleasant colonoscopic procedure (28). We did not collect information on antibiotic use in the preceding 6 mo. We also acknowledge that we did not standardize dietary intake or physical activity levels prior to each of the study visits (in order to increase study acceptability for participants) so we cannot exclude that differences in gastrointestinal transit time and absorption related to meal composition contributed in part to the results. Finally, we recognize the risk of type 1 statistical error from the large number of analyses performed.

In summary, we report that oral dosing with ω-3 PUFAs for 28 d leads to an increase in EPA and DHA bioavailability in IF, relevant to therapeutic use of ω-3 PUFAs for ileocolonic diseases. The ileal microbiome is highly variable between and within individuals. However, a relation between abundance of *Bacteroides* and luminal EPA and DHA concentration was evident and is consistent with the emerging hypothesis that ω-3 PUFA use drives increased luminal SCFA exposure (3, 8).

## Supplementary Material

nxab113_Supplemental_File

## Data Availability

Data described in the manuscript, code book, and analytic code will be made available free of charge upon request to the corresponding author.

## Notes

This study was funded by Leeds Cares (Rays of Hope) Charity. Leeds Cares had no role in the design, analysis, or writing of this article. Fatty acid measurement was supported by the Yorkshire Experimental Cancer Medicine Centre.

Author disclosures: The authors report no conflicts of interest.

Supplemental Table 1 and Supplemental Figures 1–3 are available from the “Supplementary Data” link in the online posting of the article and from the same link in the online table of contents at https://academic.oup.com/jn.

Abbreviations used: CRC, colorectal cancer; DPA, docosapentaenoic acid; IF, ileostomy fluid; LNA, α-linolenic acid; OTU, operational taxonomic unit; PCoA, principal component analysis; PERMANOVA, permutational multivariate ANOVA (test).
