# Supplementary material for: Luminal Bioavailability of Orally Administered ω-3 PUFAs in the Distal Small Intestine, and Associated Changes to the Ileal Microbiome, in Humans with a Temporary Ileostomy
Source: J Nutr. 2021 May 24;151(8):2142–52. doi: 10.1093/jn/nxab113 (PMC8349127; doi:10.1093/jn/nxab113)
Supplement: nxab113_Supplemental_File [file nxab113_supplemental_file.pdf]

Luminal bioavailability of orally administered omega-3 polyunsaturated fatty acids in the distal small intestine, and associated changes to the ileal microbiome, in humans with a temporary ileostomy. Nana et al. **Online supplementary material**

Sample ID	LNA	EPA	DPA	DHA	LA	AA	OA	PA	SA
P1 V1	0.1	1.5	0.01	0.8	1.3	2.4	3.0	62.2	240.5
P1 V2	0.3	1.5	0.07	1.8	3.1	4.8	5.4	67.2	273.6
P1 V3	0.1	1.6	0.02	1.6	1.9	2.7	3.2	74.6	325.4
P2 V1	0.8	0.6	0.01	0.6	2.2	1.7	6.5	96.6	226.2
P2 V2	0.3	52.5	0.18	52.0	2.0	9.5	2.4	44.6	109.9
P2 V3	1.0	1.1	0.04	2.7	9.8	6.1	8.0	66.9	190.9
P3 V1	3.0	1.2	0.02	1.9	28.1	15.3	60.7	121.0	323.2
P3 V2	0.2	20.6	0.08	22.4	3.1	4.9	15.7	55.1	197.2
P4 V1	0.3	0.6	0.01	0.2	2.9	1.6	6.0	93.7	367.7
P4 V2	0.6	0.7	0.01	0.1	2.0	0.9	10.5	95.5	573.3
P4 V3	0.2	1.1	0.01	1.0	0.8	1.0	5.3	118.0	634.4
P5 V1	0.4	0.4	0.01	0.3	2.9	3.1	12.9	343.9	1067.1
P5 V2	2.3	207.1	0.79	200.5	9.7	37.2	25.7	74.2	273.1
P5 V3	0.3	0.8	0.01	0.3	1.7	0.9	7.6	76.2	461.3
P6 V1	9.2	1.1	0.10	2.2	15.8	13.5	17.7	114.1	417.0
P6 V2	0.2	34.4	0.11	28.0	4.2	10.8	6.3	53.8	208.8
P6 V3	3.0	3.6	0.07	3.9	12.9	4.5	38.6	212.6	663.0
P7 V1	0.7	0.3	0.00	0.1	36.8	0.6	9.8	49.7	187.3
P7 V2	0.7	0.5	0.01	0.1	9.0	0.8	3.8	37.6	171.7
P7 V3	0.4	1.1	0.01	1.1	2.7	1.0	3.9	43.9	198.3
P8 V1	0.5	2.0	0.08	1.0	6.0	12.7	6.4	63.1	217.6
P8 V2	0.7	1.3	0.03	0.3	2.4	5.4	7.3	86.3	352.3
P8 V3	0.4	0.9	0.04	0.7	3.9	2.3	6.5	187.4	432.1
P9 V1	1.3	1.4	0.03	1.6	15.1	4.1	12.7	105.1	344.2
P9 V2	0.6	1.4	0.05	2.6	2.6	1.7	5.2	85.0	338.8
P9 V3	1.0	47.1	0.37	51.7	7.2	11.5	37.8	441.5	1472.4
P10 V1	0.2	0.8	0.04	1.0	3.5	5.4	6.6	70.3	251.5
P10 V2	0.4	1.0	0.1	1.0	8.4	12.0	15.5	143.0	510.4
P10 V3	0.8	4.2	0.1	5.3	10.7	10.0	30.3	355.4	940.6
P11 V1	2.1	1.0	0.11	1.5	12.6	1.4	9.9	115.6	347.1
P11 V2	1.4	5.7	0.03	5.9	11.0	3.2	12.8	136.6	375.8
P11 V3	2.6	7.2	0.03	5.4	42.5	4.1	42.6	103.0	249.0

Supplementary Table 1. Absolute fatty acid concentrations in ileostomy fluid in μg/mL. P, participant number; V, visit number. LNA, alpha-linolenic acid; EPA, eicosapentaenoic acid; DPA, docosapentaenoic acid; DHA, docosahexaenoic acid; LA, linoleic acid; AA, arachidonic acid; OA, oleic acid; PA, palmitic acid; SA, stearic acid. Data for participants with a V2 peak in EPA and DHA concentration (#2, 3, 5 and 6) are shaded light grey.

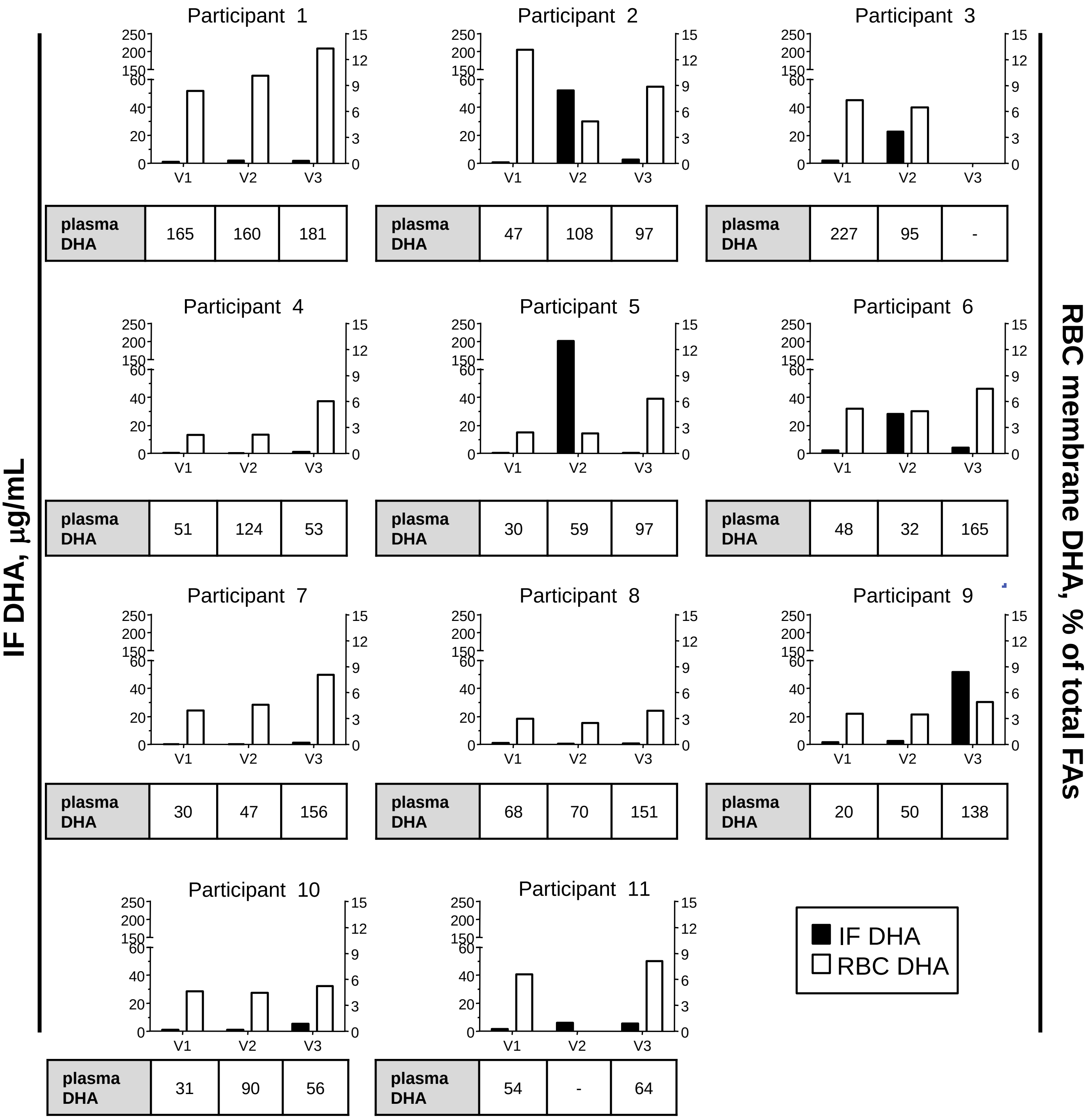

plasma DHA	V1	V2	V3
Participant 1	165	160	181
Participant 2	47	108	97
Participant 3	227	95	-
Participant 4	51	124	53
Participant 5	30	59	97
Participant 6	48	32	165
Participant 7	30	47	156
Participant 8	68	70	151
Participant 9	20	50	138
Participant 10	31	90	56
Participant 11	54	-	64

Supplementary Figure 1. Levels of DHA in ileostomy fluid (IF) as μg/mL and RBC membranes (open columns) presented as % total fatty acids, for each participant. Corresponding plasma DHA concentrations (in μg/mL) for visit (V)1-3 are below each figure panel. The timing of sample collection for V2 and V3 samples in relation to omega-3 PUFA capsules dosing is identical to that described in Figure 1 of the paper.

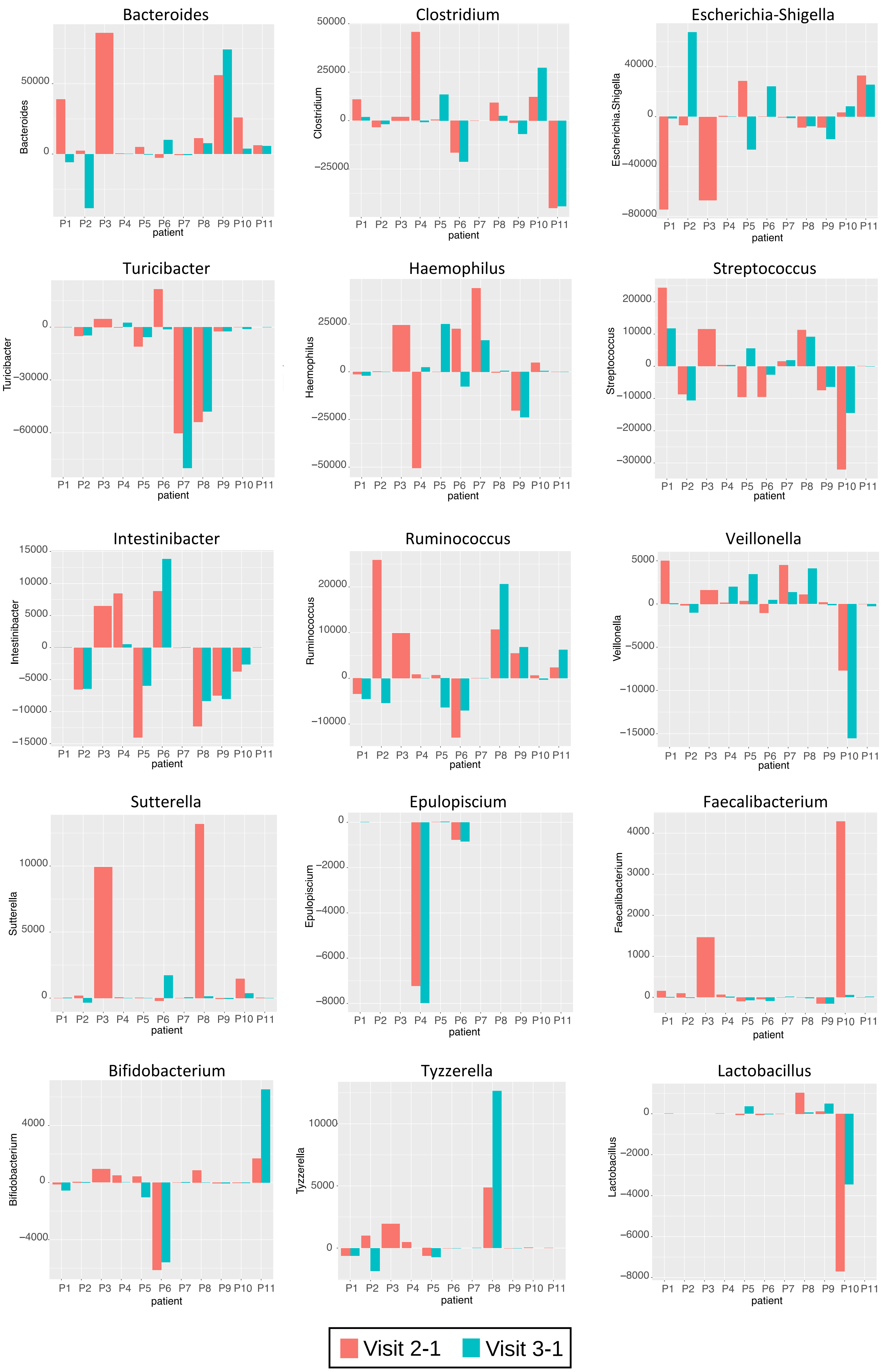

Supplementary Figure 2. Difference in operational taxonomic units (OTUs) for the top 15 genera in ileostomy fluid between visit 1 (baseline, before omega-3 PUFA supplementation) and either visit 2 (after the first dose of capsules) or visit 3 (after 28 days oral omega-3 PUFA dosing). Data are normalised to a 100,000 scale for each sample. Positive figures denote an increase compared with baseline values; negative figures denote a decrease compared with baseline values. Data are shown for all participants P1-11. There were no visit 3 data for P3.

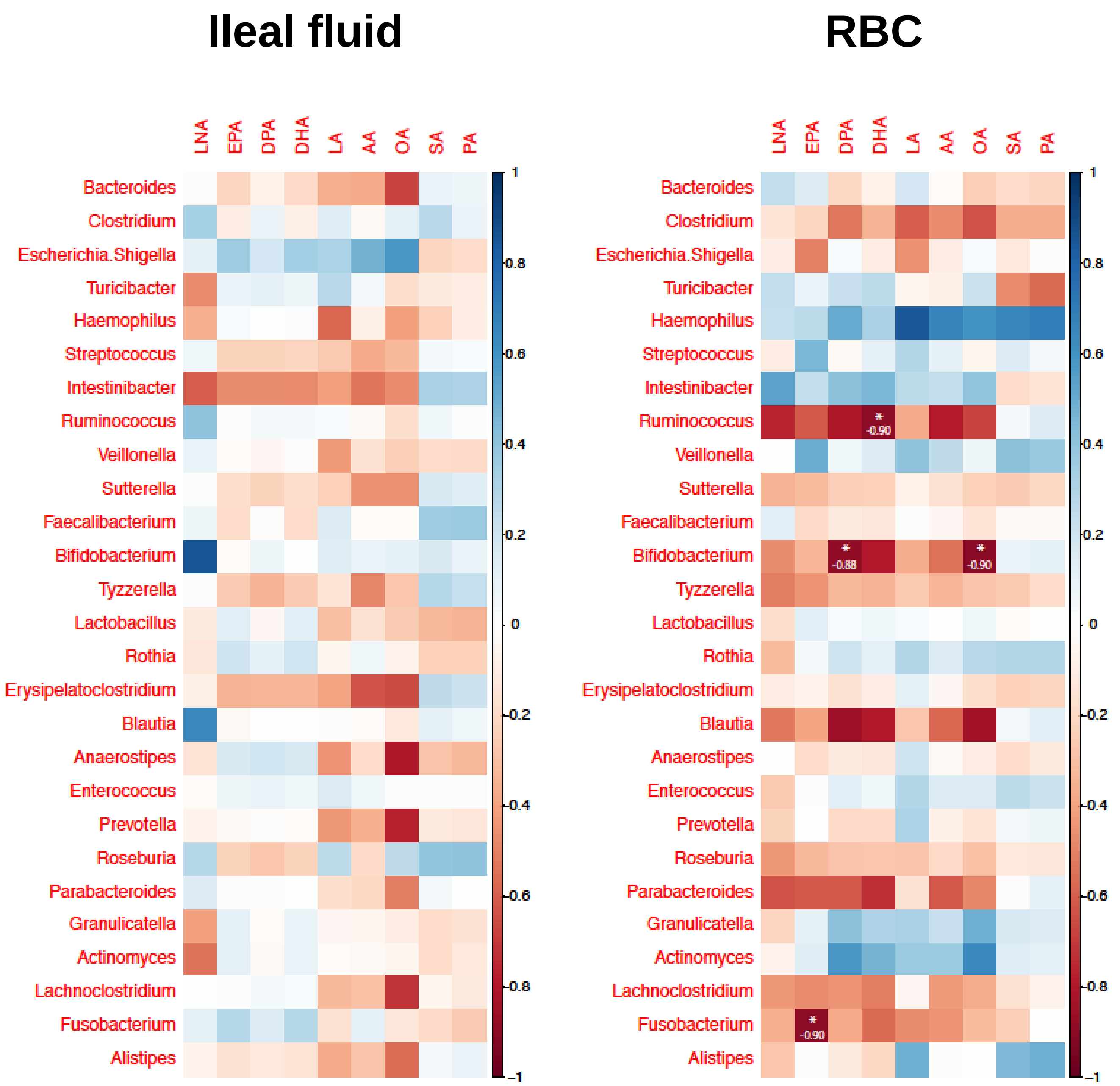

Supplementary Figure 3. Correlation matrices for the difference in bacterial genus abundance and the difference in absolute ileal fluid fatty acid levels (and red blood cell [RBC] % content) between baseline (visit [V]1) and after the first dose of omega-3 PUFA capsules (V2). LNA, alpha-linolenic acid; EPA, eicosapentaenoic acid; DPA, *n*-3 docosapentaenoic acid; DHA, docosahexaenoic acid; LA, linoleic acid; AA, arachidonic acid; OA, oleic acid; SA, stearic acid; PA, palmitic acid. Blue denotes a positive correlation. Red denotes a negative correlation. The strength of the Pearson correlation is denoted by the colour intensity (side-bar scale). Significant relationships are signified by an asterisk with the actual r^2 value. These occurred for unrelated fatty acids and RBC content changes only, not ileal fluid.
